# A predictive model for disease severity among COVID-19 elderly patients based on IgG subtypes and machine learning

**DOI:** 10.3389/fimmu.2023.1286380

**Published:** 2023-11-30

**Authors:** Zhenchao Zhuang, Yuxiang Qi, Yimin Yao, Ying Yu

**Affiliations:** ^1^ Department of Laboratory Medicine, The First Affiliated Hospital of Zhejiang Chinese Medical University (Zhejiang Provincial Hospital of Chinese Medicine), Hangzhou, China; ^2^ School of Medical Technology and Information Engineering, Zhejiang Chinese Medical University, Hangzhou, China

**Keywords:** COVID-19, elderly patients, severity, IgG subtypes, predictive model, machine learning

## Abstract

**Objective:**

Due to the increased likelihood of progression of severe pneumonia, the mortality rate of the elderly infected with coronavirus disease 2019 (COVID-19) is high. However, there is a lack of models based on immunoglobulin G (IgG) subtypes to forecast the severity of COVID-19 in elderly individuals. The objective of this study was to create and verify a new algorithm for distinguishing elderly individuals with severe COVID-19.

**Methods:**

In this study, laboratory data were gathered from 103 individuals who had confirmed severe acute respiratory syndrome coronavirus 2 (SARS-CoV-2) infection using a retrospective analysis. These individuals were split into training (80%) and testing cohort (20%) by using random allocation. Furthermore, 22 COVID-19 elderly patients from the other two centers were divided into an external validation cohort. Differential indicators were analyzed through univariate analysis, and variable selection was performed using least absolute shrinkage and selection operator (LASSO) regression. The severity of elderly patients with COVID-19 was predicted using a combination of five machine learning algorithms. Area under the curve (AUC) was utilized to evaluate the performance of these models. Calibration curves, decision curves analysis (DCA), and Shapley additive explanations (SHAP) plots were utilized to interpret and evaluate the model.

**Results:**

The logistic regression model was chosen as the best machine learning model with four principal variables that could predict the probability of COVID-19 severity. In the training cohort, the model achieved an AUC of 0.889, while in the testing cohort, it obtained an AUC of 0.824. The calibration curve demonstrated excellent consistency between actual and predicted probabilities. According to the DCA curve, it was evident that the model provided significant clinical advantages. Moreover, the model performed effectively in an external validation group (AUC=0.74).

**Conclusion:**

The present study developed a model that can distinguish between severe and non-severe patients of COVID-19 in the elderly, which might assist clinical doctors in evaluating the severity of COVID-19 and reducing the bad outcomes of elderly patients.

## Introduction

The severe acute respiratory syndrome coronavirus 2 (SARS-CoV-2) virus has given rise to a worldwide pandemic known as coronavirus disease 2019 (COVID-19). The trends of the pandemic vary among different countries and regions. Clinical experiences have shown that COVID-19 is a highly heterogeneous disease, representing a range of clinical severity, from asymptomatic and mild to severe pneumonia, acute respiratory distress syndrome (ARDS), and even death (1, 2). The first report of SARS-CoV-2 infections in the population was from China. Initial findings from China suggested that older age is associated with a higher likelihood of experiencing and suffering from COVID-19. Immunological senescence and inflammation play a severe role in contributing to older patients who are more prone to severe outcomes of COVID-19 (1, 3).

IgG antibodies, also known as immunoglobulin G, offer a prominent means of protection against contagious illnesses. Antigen–IgG immune complexes could be formed when IgG antibodies bind directly to pathogens. During an infection, the inflammatory response is directed by these complexes of the immune system. Following viral infection, the initiation of IgG-mediated effector control happens as reactive antibodies bind to viral particles (4). Chakraborty et al. (5) found that a greater number of individuals with severe COVID-19 have increased levels of particular pro-inflammatory antibody variants. These variants are identified by the presence of IgG 3 and IgG 1 antibodies with F0N0 glycoform modification.

Data mining algorithms and predictive analysis are the theoretical core of machine learning analysis, which is to identify individual features of data from machine learning, establish models through science, and subsequently utilize new data through these models to forecast future data (6). Machine learning (ML) is of great value in medical research and a number of studies have utilized machine learning as a tool that can be used to predict COVID-19 (7–10). Nevertheless, some studies require medical imaging such as CTs and X-rays, and the parameters are relatively complex, and the influences of ionizing radiation are unavoidable (11, 12). In addition, there is currently a lack of prediction models that consider IgG subtypes in COVID-19 patients, with the majority of existing models concentrating on the severity of the disease in ordinary individuals rather than the elderly population (13, 14). **Table 1** summarizes recent work on COVID-19 by machine learning algorithms.

**Table 1:** Survey on existing machine learning algorithms.

Source, y	Task	Data Source and Size	ML Model	Evaluation of Model Performance	Independent External Validation
Moulaei et al. (7), 2022	Prediction of COVID-19 mortality	Retrospective analysis of 1,500 patients with COVID-19 in a single center	RF, XGBoost, KNN, MLP, logistic regression, J48 DT, naive Bayes	Accuracy, sensitivity, precision, specificity, and AUC	No external validation set
Shahin et al. (8), 2022	Detection and classification of COVID-19 virus	Retrospective analysis of 122 patients	Naive Bayes Classification, DT, SVM, RBF, and K-means clustering	Accuracy, correctness, recall, and F1 score	No external validation set
Alali et al. (9), 2022	prediction of COVID−19 spread	Retrospective study of a dataset from CSSEGISand-Data	GPR, SVR, Boosted trees, bagged trees, DT, RF, and XGBoost	Root mean square error, mean absolute error, and mean absolute percentage error	No external validation set
Pan et al. (10), 2020	Prognostic assessment of COVID-19	Retrospective analysis of 123 Patients with COVID-19 in the ICU in a single center	AdaBoost, GBDT, XGBoost, CatBoost	AUC, threshold, youden index, p-value of the AUC, accuracy, sensitivity, specificity, PPV, NPV, PLR, and NLR	No external validation set
Budimirovic et al. (11), 2022	Prediction of COVID-19 severity	Infected lung images from a large dataset	MWOA-SSA, MWOA, PCA	sensitivity, specificity, accuracy, PPV, F1-score, and NPV	No external validation set
Zivkovic et al. (12), 2022	COVID-19 early diagnostics from X-ray images	Images from the COVID-19 radiography database	CNN, XGBoost	Accuracy, precision, recall, F1 score	No external validation set

AdaBoost, adaptive boosting; GBDT, gradient boosting decision tree; XGBoost, eXtreme gradient boosting; CatBoost, categorical boosting; AUC, the area under the curve; PPV, positive predictive value; NPV, negative predictive value; PLR, positive likelihood ratio; NLR, negative likelihood ratio; DT, decision trees; SVM, support vector machine; RBF, radial basis function; RF, random forest; KNN, k-nearest neighborhood; MLP, multi-layer perceptron; SVR, support vector regression; GPR, Gaussian process regression; MWOA, a modified whale optimization algorithm; MWOA-SSA, a modified whale optimization algorithm with the salp swarm algorithm; PCA, principal component analysis; CNN, convolutional neural network.

As age increases, the probability of infection and the mortality rate of COVID-19 also increased. The elderly are particularly vulnerable to COVID-19 infection due to their weakened immune systems and the presence of other chronic diseases such as hypertension and diabetes. This study question thus highlights the therapeutic significance of early identification of COVID-19-related fatalities in elderly people. Because the immune response has such a large influence, it is also important to investigate immunological antibodies to distinguish between non-severe and severe COVID-19 instances and to provide unique treatment approaches.

Therefore, this study developed a model utilizing IgG subtypes and machine learning to help clinicians distinguish the severity of COVID-19 in elderly individuals and implement effective interventions to reduce mortality. In the present study, the major contributions are as follows:

1. A novel model for predicting the severity of COVID-19 based on IgG subtypes and machine learning is developed.
2. This study focuses on elderly patients over the age of 60 rather than ordinary individuals.
3. In this study, five machine learning algorithms are compared to predict the severity of elderly COVID-19 patients, and the logistic regression model demonstrated the highest prediction performance among them.

The structure of this research has been organized as follows. *Section 2* shows the methods including patient involvement and dataset selection. *Section 3* presents screening variables and optimal machine learning models to predict the severity of COVID-19 in elderly patients. *Section 4* discusses the results. *Section 5* discusses the limitations. *Section 6* summarizes the article and the prospect of the next step.

## Materials and methods

### Ethics statement

This study was approved by the Ethics Committee of the First Affiliated Hospital of Zhejiang Chinese Medical University with approval number 2023-KLS-034-01.

### Patient involvement

According to the standards of the China Novel Coronavirus Infection Diagnosis and Treatment Program (Trial 10th Edition) and the clinicians’ diagnoses (15), we conducted a search for patients of non-severe and severe COVID-19 (age ≥60 years) diagnosed from 1 to 16 January 2023, in Zhejiang Provincial Hospital of Chinese Medicine (Hubin). Two groups were formed for the elderly patients with COVID-19, namely, non-severe and severe groups. Included in the study were a combined total of 41 cases classified as non-severe and 62 cases classified as severe. Patients in the severe group progressed to severe or critical COVID-19 or pneumonia-related deaths while hospitalized, whereas patients in the non-severe group remained in non-severe states (mild or moderate COVID-19) while hospitalized. Furthermore, 22 cases from Zhejiang Provincial Hospital of Chinese Medicine (Qiantang and Xixi) were collected as an external validation cohort from 1 to 16 January 2023.

Mild pneumonia with respiratory tract infection, such as dry throat, sore throat, cough, and fever, was the main manifestation. Imaging findings show characteristics of COVID-19 pneumonia, and abnormal clinical symptoms can be observed in moderate pneumonia. Patients are determined to have severe pneumonia if they meet any of the following criteria (1): a notable rise in respiration rate, with RR  ≥30/min; (2) oxygen saturation of 93% or lower while at rest; (3) a PaO_2_/FiO_2_ ratio of 300 mmHg or less (1 mmHg = 0.133 kPa); and (4) significant advancement of pulmonary lesions by more than 50% within 24–48 h, as observed through pulmonary imaging. Critical pneumonia occurs when the disease progresses rapidly with any of the following criteria: (1) respiratory insufficiency requiring mechanical ventilation, (2) shock, and (3) a combination of organ failure and monitoring in the ICU setting. The exclusion criterion was other viral pneumonia.

### Data collection

Detailed information on the baseline population characteristics (age, gender, and comorbidities) and clinical laboratory data of these patients were meticulously gathered from their electronic medical records. Laboratory data include routine blood examinations, C-reactive protein, coagulation indicators, cytokines, and IgG subtypes. After enrollment, 103 elderly individuals were randomly assigned to the training cohort (80%) and the testing cohort (20%). By setting a random seed (random seed=1), the present study can ensure the repeatability of the random process, allowing us to accurately reproduce research results when needed. The best model hyperparameters selected were by grid search and carried out fivefold cross-validation. In the fivefold cross-validation, the dataset was split into five parts of approximately equal size: one of the five parts for testing and the remaining four parts for training. Fivefold cross-validation was cycled through the process five times. The models were constructed in the training cohort using laboratory tests and machine learning techniques and subsequently verified in the testing cohort. The external validation cohort was validated against the final filtered-out optimal model.

### Statistical analysis

Analyses were performed utilizing SPSS 26.0 and R 4.3.1 software. Frequencies and percentages were used to present categorical variables, while mean ± standard deviation or median and interquartile range (IQR) were used for continuous variables. The χ^2^ test was used to analyze count data, while independent samples *t*-test or Wilcoxon test were used to analyze measurement data.

Significant differences between severe and non-severe groups were identified through a univariate analysis, followed by the utilization of least absolute shrinkage and selection operator (LASSO) regression to select the factors associated with COVID-19 severity. By cohort seed, we selected 80% of the patients for deriving the optimal model (training cohort), whereas the other 20% of patients were allocated to the validation cohort. Subsequently, the present study established predictive models using meaningful factors identified through LASSO regression. In both the training and validation cohorts, calibration plots were utilized to graphically evaluate calibration, while a receiver operating characteristic (ROC) curve and the area under the ROC curve (AUC) were employed to assess calibration. The interpretation of the feature ranking was done using Shapley additive explanations (SHAP) plots. Statistical significance was determined by considering a p-value<0.05.

### Machine learning

For the development of an ML-based algorithm, the Deepwise & Beckman Coulter DxAI platform utilized an online statistics tool. The platform has the capability to automatically select machine learning models, display the analysis data and generate a page of analysis online.

## Results

### Demographic characteristics

The present study first compared IgG subtypes between COVID-19 elderly patients and healthy individuals 60 years of age and older. As can be seen in **Table 2**, there were significant differences in four subtypes of IgG between the two groups (p<0.05).

**Table 2:** Comparison of IgG subtypes between elderly COVID-19 patients and healthy individuals.

Variable category	COVID-19 elderly patients (*n* =103)	Healthy elderly individuals (*n* =40)	P*-*value
IgG 1(μg/mL)	5,965.00(3,584.00–10,259.00)	8,265.00(7,364.00–9,452.75)	0.001
IgG 2(μg/mL)	2,874.00(1,834.00–4,331.00)	3,667.00(2,700.75–4,763.00)	0.004
IgG 3(μg/mL)	227.00(128.00–444.00)	536.00(338.00–645.25)	<0.001
IgG 4(μg/mL)	336.00(152.00–652.00)	594.00(255.75–863.00)	0.017

In order to conduct a more in-depth investigation, this study explored the distribution of IgG subtypes among elderly COVID-19 patients, distinguishing between those with severe symptoms and those with non-severe symptoms. The demographic characteristics of these patients are summarized in **Table 3**. This study consisted of 41 (39.81%) classified as non-severe and 62 (60.19%) classified as severe. There were 43 men (69.35%) and 19 women (30.65%) in the severe group, while there were 22 men (53.66%) and 19 women (46.34%) in the non-severe group. As shown in **Table 3**, there were no statistical differences in the non-severe and severe groups by gender (p=0.106 >0.05), which was comparable. In terms of age, the severe group had a significantly higher mean age compared to the non-severe group (median, 84.50: 75.00), with a highly significant difference between the two groups (p<0.001). Older men had a significantly higher rate of severe COVID-19 compared to women. The present research aligns with the findings reported by Jin et al. (16), who described worse outcomes and deaths in men with COVID-19. The most prevalent comorbidity among severe patients was hypertension (66.13%), followed by diabetes (32.26%). Additionally, coronary heart disease, anemia, tumors, and COPD were present in 20.97%, 16.13%, 12.90%, and 9.68% of severe patients, respectively.

**Table 3:** Baseline characteristics of COVID-19-infected patients.

Characteristic	Non-severe Patients (*n* =41)	Severe Patients (*n* =62)	Validation Cohort (*n* =22)	P*-*value
Gender				
Male[(n, %)]	22 (53.66)	43 (69.35)	15 (68.18)	0.106
Female[(n, %)]	19 (46.34)	19 (30.65)	7 (31.82)	
Age [year (median IQR)]	75.00 (67.00–83.50)	84.50 (74.25–88.00)	75.00 (71.00–81.00)	<0.001
Basic disease				
Yes^a^[(n, %)]	29 (70.73)	51 (82.26)	19 (86.36)	0.169
No[(n, %)]	2 (29.27)	11 (17.74)	3 (13.64)	
Comorbidities [(n, %)]				
Tumor^b^	6 (14.63)	8 (12.90)	1 (4.55)	0.802
Hypertension	24 (58.54)	41 (66.13)	12 (54.55)	0.443
Diabetes	10 (24.39)	20 (32.26)	5 (22.73)	0.390
COPD	2 (4.88)	6 (9.68)	4 (18.18)	0.373
Anemia	3 (7.32)	10 (16.13)	2 (9.09)	0.187
Coronary heart disease	3 (7.32)	13 (20.97)	4 (18.18)	0.061

a Patients with one of the following: tumor, hypertension, diabetes, COPD, anemia, or coronary heart disease.

b Any type of tumor.

COPD, chronic obstructive pulmonary disease.

In an external validation cohort, this study consisted of 11 (50.00%) classified as non-severe and 11 (50.00%) classified as severe. In the group, 15 patients (68.18%) were male, and 7 (31.82%) were female. The median age of this group was 75. The most prevalent comorbidity among patients was hypertension (54.55%), followed by diabetes (22.73%). Additionally, coronary heart disease, COPD, anemia, and tumors were present in 18.18%, 18.18%, 9.09%, and 4.55% of patients, respectively.

### Comparison of biomarkers between non-severe and severe COVID-19 patients

During the process of comparing the two biomarkers, the present study included each subtype of IgG and made pairwise ratios, which were also compared to IgG Sum, yielding several new indicators. As shown in **Table 4**, except for IgG 1/IgG 4, LY #, and HGB, the severe COVID-19 group exhibited significantly elevated levels of IL-2, IL-6, IgG 2/IgG 1, IgG Sum/IgG 1, IgG 2/IgG Sum, CRP, PT, INR, DD, WBC, NE #, NLR, RDW, and PDW in comparison to the non-severe COVID-19 group (p<0.05).

**Table 4:** Comparison of biomarkers between non-severe and severe COVID-19 patients.

Variable category	Non-Severe elderly patients (*n* =41)	Severe elderly patients (*n* =62)	P*-*value
IL-2(pg/ml)	0.86 (0.81–0.96)	0.96 (0.86–1.45)	0.003
IL-4(pg/ml)	1.31 (1.06–1.49)	1.22 (1.06–1.49)	0.484
IL-6(pg/ml)	6.39 (3.13–25.97)	23.47 (7.98–76.88)	<0.001
IL-10(pg/ml)	3.06 (1.92–4.68)	3.65 (2.71–5.97)	0.051
TNF-α(pg/ml)	1.16 (0.91–1.55)	1.29 (0.93–3.50)	0.146
IFN-γ(pg/ml)	1.28 (0.92–1.79)	1.38 (1.12–4.37)	0.161
IgG 1(μg/mL)	6,645.00(4,075.50–11,488.50)	5,614.50(3,389.25–8,213.75)	0.129
IgG 2(μg/mL)	2,526.00(1,627.50–3,999.00)	2,980.50(1,899.75–4,624.75)	0.218
IgG 3(μg/mL)	203.00 (121.00–480.50)	247.50(132.50–433.25)	0.869
IgG 4(μg/mL)	319.00 (138.00–663.00)	372.00(167.50–643.25)	0.433
IgG Sum(μg/mL)	11,797.88 ± 6,238.26	10,792.35 ± 5,625.25	0.397
IgG 1/IgG 3	26.73 (17.77–43.41)	25.30 (15.97–34.20)	0.269
IgG 2/IgG 1	0.34 (0.26–0.54)	0.52 (0.33–0.73)	0.007
IgG 1/IgG 4	21.77 (11.55–47.78)	17.09 (7.47–30.56)	0.047
IgG 2/IgG 3	11.82 (7.58–19.79)	12.14 (7.48–20.48)	0.824
IgG 2/IgG 4	7.71 (4.23–16.92)	8.15 (5.35–12.83)	0.909
IgG 3/IgG 4	0.59 (0.38–2.50)	0.59 (0.28–1.43)	0.374
IgG Sum/IgG 1	1.47 (1.34–1.70)	1.66 (1.49–1.83)	0.009
IgG 2/IgG Sum	0.26 ± 0.09	0.31 ± 0.10	0.017
IgG 3/IgG Sum	0.024 (0.016–0.035)	0.024 (0.016–0.036)	0.628
IgG 4/IgG Sum	0.029 (0.015–0.060)	0.040 (0.023–0.065)	0.147
CRP(mg/L)	20.40 (5.10–51.54)	56.44 (24.81–100.60)	0.001
PT(s)	11.70 (11.20–12.60)	12.75 (11.50–14.25)	0.001
INR	0.98 (0.94–1.06)	1.08 (0.97–1.20)	0.002
FIB(g/L)	4.09 (2.90–5.36)	4.21 (3.26–5.37)	0.515
TT(s)	17.70 (17.10–18.85)	17.60 (16.83–19.30)	0.898
APTT(s)	29.00 (26.70–34.15)	31.55 (28.90–35.58)	0.067
DD(mg/l)	0.71 (0.41–1.08)	1.62 (0.71–5.61)	<0.001
WBC(×10^9^/L)	6.00 (4.05–7.40)	7.95 (5.50–10.73)	0.011
NE #(×10^9^/L)	4.10 (2.50–5.30)	6.50 (4.23–9.25)	<0.001
LY #(×10^9^/L)	1.00 (0.60–1.50)	0.50 (0.40–0.83)	<0.001
NLR	4.30 (2.47–6.23)	12.76 (5.54–22.48)	<0.001
MO #(×10^9^/L)	0.50 (0.30–0.70)	0.50 (0.28–0.80)	0.704
EO #(×10^9^/L)	0.01 (0.00–0.04)	0.01 (0.00–0.02)	0.351
BA #(×10^9^/L)	0.01 (0.01–0.03)	0.01 (0.01–0.02)	0.157
RBC(×10^12^/L)	3.83 (3.28–4.30)	3.75 (3.10–4.08)	0.091
HGB(g/L)	119.59 ± 20.33	110.10 ± 21.73	0.028
HCT(%)	34.74 ± 5.87	32.15 ± 6.37	0.040
MCV(fl)	90.30 (88.45–93.90)	91.50 (86.70–96.90)	0.944
MCH(pg)	31.81 ± 2.73	31.56 ± 2.96	0.660
MCHC(g/L)	344.37 ± 9.27	342.58 ± 10.11	0.367
RDW(%)	13.20 (12.85–13.75)	14.05 (13.30–15.22)	0.003
PLT(×10^9^/L)	197.00 (137.00–295.00)	158.50(115.75–232.00)	0.116
MPV(fl)	9.30 (8.25–10.50)	9.50 (8.88–10.63)	0.361
PDW(%)	16.88 ± 0.63	17.35 ± 0.76	0.001

IL-2, interleukin 2; IL-4, interleukin 4; IL-6, interleukin 6; IL-10, interleukin 10; TNF-α, tumor necrosis factor α; IFN-γ, interferon γ; IgG, immunoglobulin G; IgG Sum, the sum of four categories of IgG; CRP, C reactive protein; PT, prothrombin time; INR, international normalized ratio; FIB, fibrinogen; TT, thrombin time; APTT, activated partial thromboplastin time; DD, D-dimer; WBC, leukocyte count; NE #, neutrophil; LY #, lymphocyte; NLR, neutrophil to lymphocyte ratio; MO #, monocyte; EO #, eosinophil; BA #, basophil; RBC, red cell count; HGB, hemoglobin; HCT, hematocrit; MCV, average red blood cell volume; MCH, average red blood cell hemoglobin content; MCHC, average red blood cell hemoglobin concentration; RDW, red blood cell volume distribution width; PLT, platelet; MPV, average platelet volume; PDW, platelet volume distribution width.

### The correlation between biomarkers and COVID-19 severity in two groups

**Figure 1:**
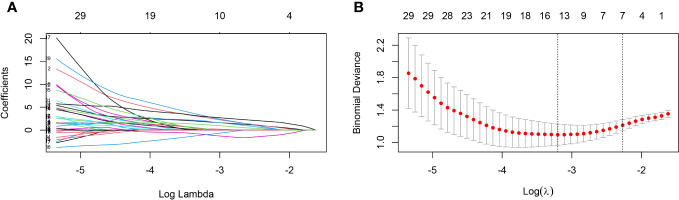
Predictors selection using LASSO regression analysis and 10-fold cross-validation. **(A)** Bias selection of the tuning parameter (lambda) in LASSO regression based on the minimum standard (left dashed line) and 1-SE standard (right dashed line). **(B)** A joint plot was created based on the log-likelihood. In this study, the selection of predictive factors was based on the 1-SE standard (right dashed line), resulting in the selection of seven non-zero factors. LASSO, least absolute shrinkage and selection operator; SE, the standard error.

The present study collected 46 features from elderly individuals diagnosed with COVID-19, and after excluding unrelated and redundant features, 18 features were retained for LASSO regression analysis. To screen for factors associated with the severity of COVID-19, an analysis using LASSO regression was conducted. The results of 103 elderly patients showed that age, IL-2, IgG Sum/IgG 1, DD, LY #, NLR, and PDW were considered to be relevant factors affecting the severe degree of COVID-19 (**Figure 1**). Additionally, the present study generated correlation heatmaps and determined feature importance using the correlation factors chosen through LASSO regression.

### Areas under ROC

**Figure 2:**
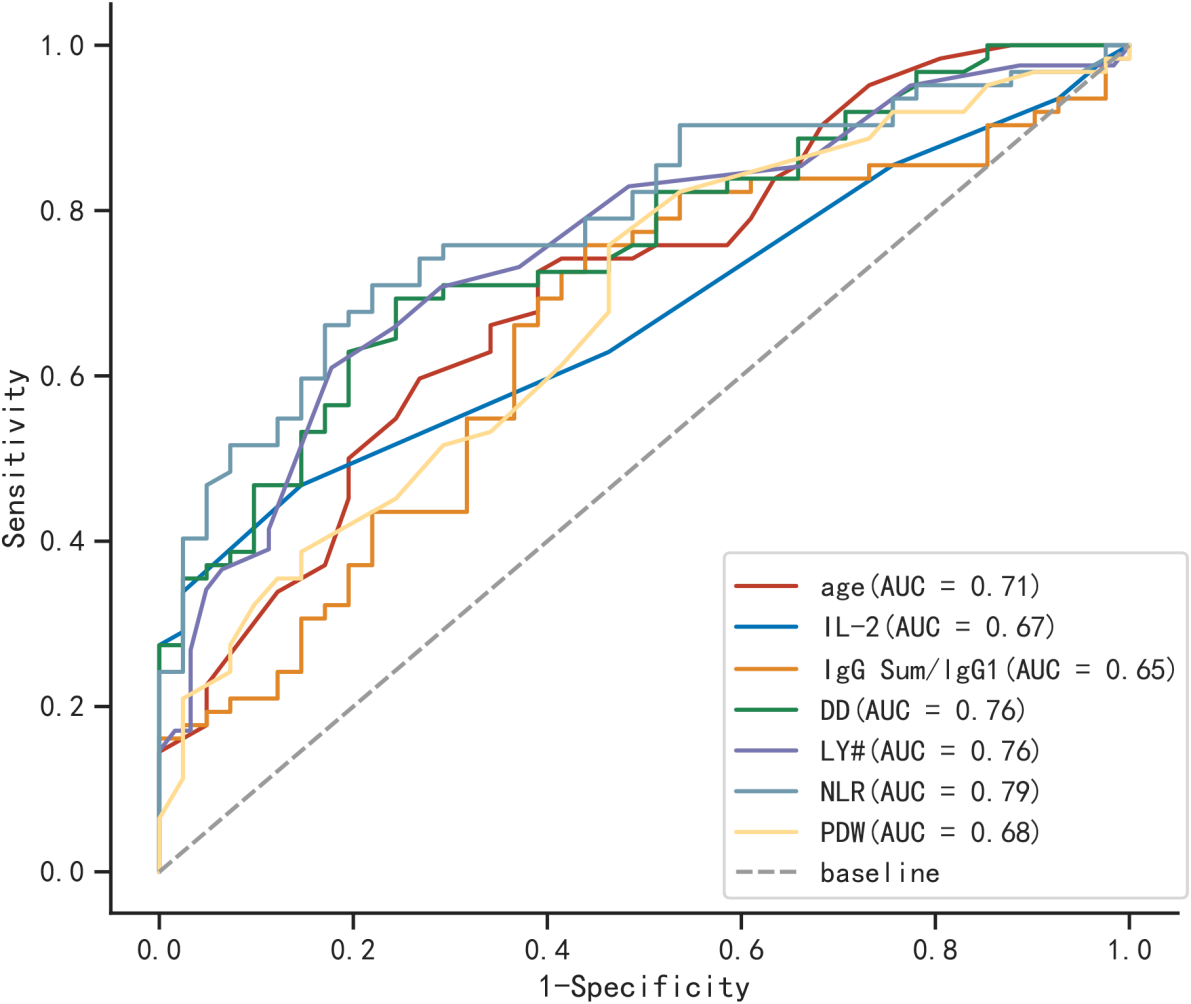
ROC curves for different biomarkers in predicting severe COVID-19 elderly patients.

In **Figure 2**, the ROC curves and AUC are depicted, representing various biomarkers with significant differences between the two groups in predicting severe COVID-19 elderly patients. Among them, NLR was the most efficient of these (AUC=0.790), followed by DD and LY # (AUC=0.760).

### Correlation heatmaps and feature importance of biomarkers

**Figure 3:**
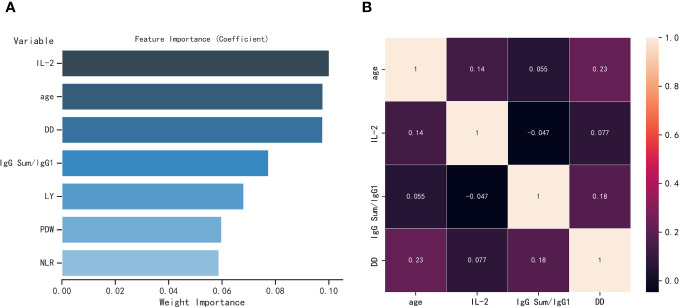
**(A)** Feature importance of seven parameters selected by LASSO regression. **(B)** Heatmap of correlation of four parameters, where one variable is plotted on the x-axis and the other on the y-axis for both severe elderly and non-severe elderly patients; antique white for positive correlation and black for negative correlation.

After analyzing the importance of various features, the present study ultimately selected four indicators based on the number of elderly individuals affected by COVID-19. The feature importance between age, IL-2, IgG Sum/IgG 1, DD, LY, PDW, and NLR are shown in **Figure 3A**. Age, IL-2, IgG Sum/IgG 1, and DD are the top 4 of the seven indicators. Then, the correlations among four individual indicators are examined. As shown in **Figure 3B**, age, IL-2, IgG Sum/IgG 1, and DD showed a low correlation, which could prevent the model from overfitting.

### Comparison of machine learning algorithms and identification of the optimal model

The AUCs of five machine learning algorithms for fivefold cross-validation on the training cohort are shown in **Table 5**. In the testing cohort, the results of five machine learning algorithms show AUCs of 0.735 for eXtreme gradient boosting (XGBoost), 0.866 for logistic regression, 0.781 for random forest, 0.812 for adaptive boosting (AdaBoost), and 0.856 for support vector machines (SVMs). The logistic regression model demonstrated the highest prediction performance among these models.

**Table 5:** Diagnostic efficacy of five classifiers in the training and testing cohorts for fivefold cross-validation.

Classifier	Cohorts	AUC	Cutoff	Accuracy	Sensitivity	Specificity	Positive predictive value	Negative predictive value	F1
XGBoost	Training	1.000	0.730	0.988	1.000	1.000	1.000	0.969	1.000
	Testing	0.735	0.730	0.705	0.679	0.842	0.765	0.629	0.712
Logistic regression	Training	0.875	0.529	0.817	0.779	0.894	0.910	0.731	0.839
	Testing	0.866	0.529	0.781	0.773	0.935	0.875	0.626	0.814
Random Forest	Training	1.000	0.590	0.976	0.996	0.994	0.996	0.948	0.996
	Testing	0.781	0.590	0.743	0.821	0.772	0.754	0.746	0.783
AdaBoost	Training	1.000	0.508	0.988	1.000	1.000	1.000	0.972	1.000
	Testing	0.812	0.508	0.638	0.717	0.920	0.830	0.407	0.756
SVM	Training	0.768	0.613	0.712	0.701	0.759	0.805	0.616	0.749
	Testing	0.856	0.613	0.705	0.720	0.980	0.832	0.635	0.762

XGBoost, eXtreme gradient boosting; AdaBoost, adaptive boosting; SVM, support vector machines.

### Analysis and assessment of machine learning model

**Figure 4:**
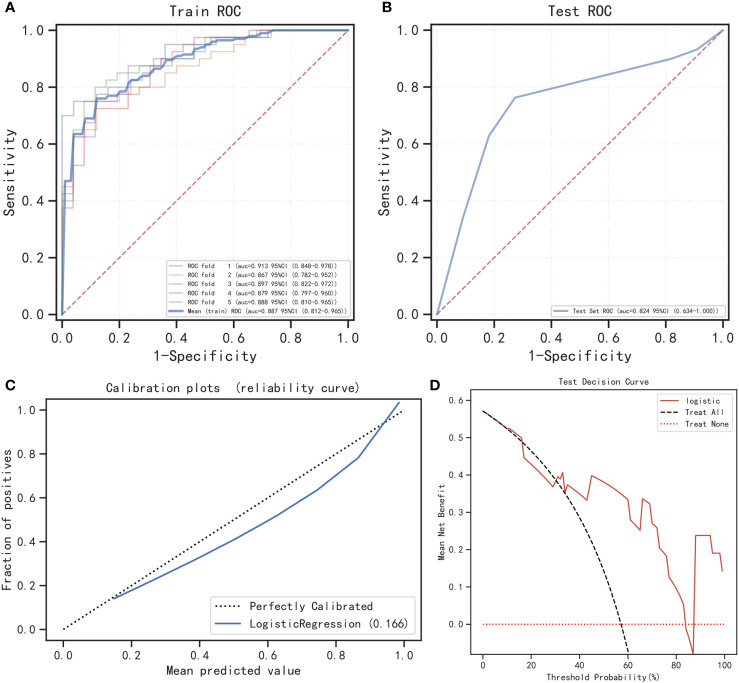
Performance of the prediction model. **(A)** The training cohort’s ROC curve; **(B)** the testing cohort’s ROC curve; **(C)** calibration curve analysis; **(D)** decision curve analysis.

On the basis of the results shown in **Table 6** and **Figure 4**, it can be observed that the logistic regression model exhibited a strong discriminatory ability in distinguishing between two groups. In the testing cohort, the model demonstrated AUC, accuracy, specificity, and positive predictive value exceeding 80% (**Figures 4A, B**). Moreover, the calibration curve demonstrated a strong correlation between actual and predicted probabilities, indicating excellent calibration of the model. According to **Figures 4C, D**, the DCA curve indicated a strong clinical benefit of the model.

**Table 6:** Diagnostic efficacy of logistic regression model in the training and testing cohorts for fivefold cross-validation.

Cohorts	AUC	Cutoff	Accuracy	Sensitivity	Specificity	Positive predictive value	Negative predictive value	F1
Training cohort	0.889	0.602	0.802	0.780	0.876	0.908	0.700	0.837
Testing cohort	0.824	0.543	0.81	0.75	0.889	0.833	0.778	0.789

**Figure 5:**
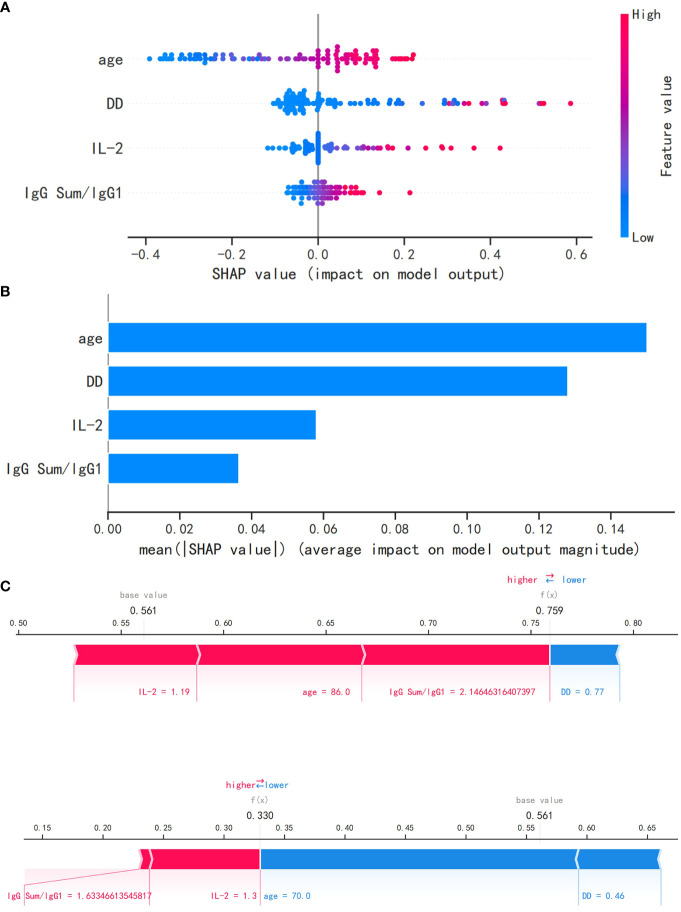
The logistic regression model utilizing the SHAP algorithm. **(A)** The SHAP value, which indicates the level of impact on the result, is represented on the abscissa for each feature. A sample is represented by each dot. As the color becomes more red, the feature’s value increases, while a bluer color indicates a lower value. **(B)** The SHAP analysis revealed the ranking of feature importance. IL-2, interleukin 2; SHAP, Shapley additive explanations. **(C)** The SHAP force plot for severe patients with COVID-19. **(D)** The SHAP force plot for non-severe patients with COVID-19.

**Figure 5A** shows the relationship between the observed values of the four most relevant features that we selected and the SHAP values. As shown in **Figure 5B**, the logistic regression model interpretation of feature ranking, as per the SHAP algorithm, indicates that age, DD, IL-2, and IgG Sum/IgG 1 were the most influential characteristics for predicting outcomes of elderly patients. The greater the mean absolute Shapley value of the features, the greater the importance of the clinical features for the model prediction. Using SHAP force plots, the study can visualize the Shapley value for each feature as a force that increases (positive) or decreases (negative) its baseline predicted value. **Figure 5** shows the individual force plots for severe patients with COVID-19 (**Figure 5C**) and non-severe patients with COVID-19 (**Figure 5D**). The probabilistic predicted value of the severe group was 0.759. The positive contribution value features in red represent pushing up the model score, while the negative contribution features in blue represent pushing down the model score. The length of the arrow helps to visualize the extent of the impact on the prediction. The longer the arrow, the greater the impact on the prediction of COVID-19 severity.

### External validation of logistic regression model

**Figure 6:**
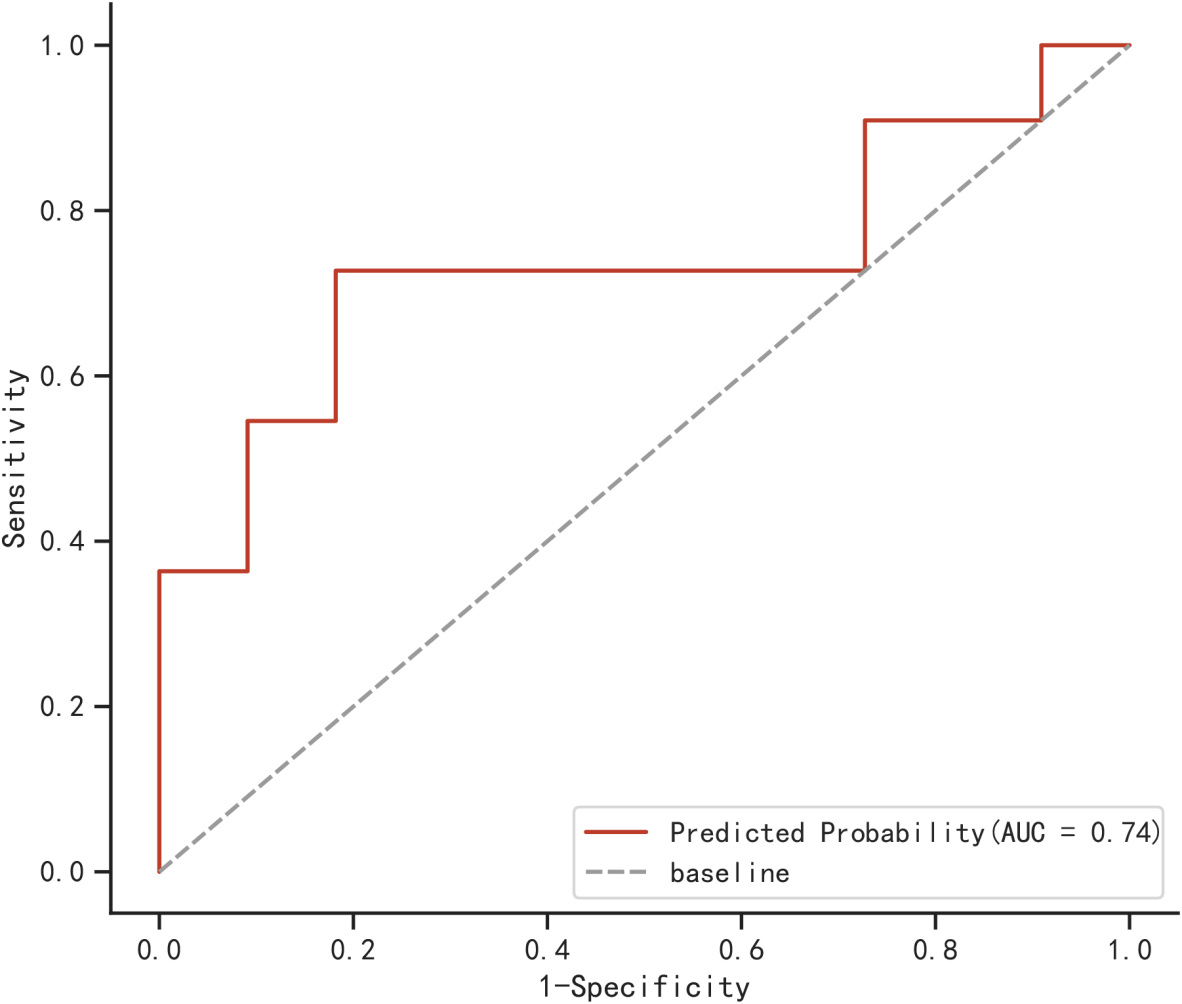
ROC for external validation of logistic regression model.

A total of 22 elderly COVID-19 patients were collected from other two centers as an external validation cohort. The AUC of the newly built model was 0.74, as demonstrated in **Figure 6**, using the validation cohort from an external source.

### Compared with different levels of clinicians

**Figure 7:**
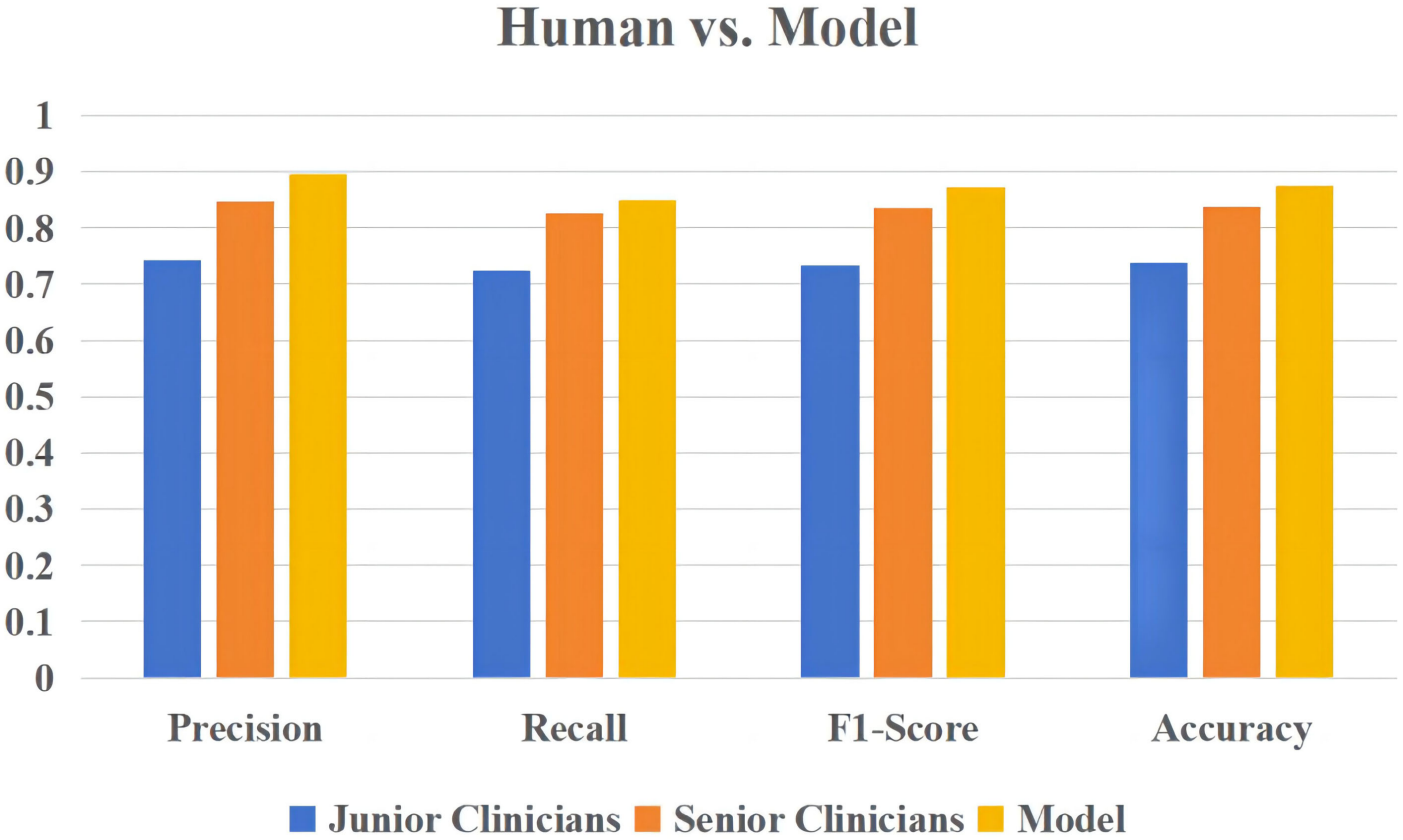
The overall performance of the logistic regression model versus human diagnosis in predicting the severity of elderly COVID-19 patients.

Using a logistic regression model, the present study compared the performance of four clinicians (including two junior clinicians and two senior clinicians) in predicting the severity of elderly COVID-19 patients. **Figure 7** demonstrates the performance comparison between the logical regression model and the human diagnosis of elderly COVID-19 patients. Among the results, the logistic regression model had an accuracy rate of 0.875, which is significantly higher than that of senior clinicians (0.8375) and junior clinicians (0.7375). The newly built model also performed better than human classifiers in terms of F1-score, recall, and precision.

## Discussion

COVID-19 is spreading throughout the world at a high speed. Although the majority of individuals have modest symptoms and a favorable prognosis, COVID-19 could progress to ARDS and possibly death. The risk of contracting COVID-19 is higher among the elderly, and they experience more severe symptoms compared to other age groups (17, 18). Effective COVID-19 treatments are still lacking (19, 20). Currently, several models have been suggested for forecasting the severity of COVID-19, with the majority concentrating on ordinary patients, while limited emphasis has been placed on elderly patients (13, 21, 22). Therefore, a predictive model for monitoring disease progression and forecasting the severity of COVID-19 in elderly individuals is urgently needed.

In recent years, machine learning has been developing rapidly, which has been widely used in predicting human diseases (23, 24), recognizing medical images (25, 26), and analyzing clinical laboratory data (27). ML can help humans efficiently process large amounts of clinical data and look for connections between different laboratory results. As medical laboratory practitioners, what are we trying to do through machine learning to help clinicians differentiate the severity of elderly COVID-19 patients?

In this study, age, IL-2, IgG Sum/IgG 1, and DD were identified and utilized in the development of the model. Through evaluation using the AUC value, calibration plot, and DCA plot, the model demonstrated good discrimination and calibration in predicting severe and non-severe cases of COVID-19 in elderly patients. This indicates a strong performance and higher clinical utility. Furthermore, the model performed effectively in both the testing cohort (AUC=0.824) and the external validation cohort (AUC=0.74). These results indicated that the model had significant value in accurately evaluating the probability of severe COVID-19 occurring in elderly patients on an individual basis.

Patients with comorbidities have been shown to be more likely to present with severe pneumonia (28). The present study found no statistically significant difference in tumor, diabetes, hypertension, coronary heart disease, COPD, and anemia between the two groups of different severity (p >0.05). In this study, 63.11% of patients were male, which was similar to the proportion of men (67.68%) reported by Chen et al. (2). Additionally, it was observed that severe patients tended to be significantly older compared to non-severe patients.

Among the common laboratory abnormalities, this study observed an increased total leukocyte count, increased NE #, and decreased LY # in severe patients. Pneumonia progression in elderly individuals with COVID-19 was influenced by elevated NLR and age, as reported in a study (29). This corresponds with the results of the present study. The differences in NE #, LY #, and NLR were statistically significant compared with the non-severe group (p<0.001), while the difference in total leukocyte count was statistically significant (p =0.011<0.05). RDW reflects the level of a size change between red blood cells; Lee et al. (30) found a potential association between it and the risk of death in COVID-19 patients, while the present study reveals that RDW was greater in severe elderly patients compared to non-severe individuals (median, 14.05: 13.20, p<0.01) and also suggest that elevated RDW levels are associated with adverse outcomes in elderly patients. Interestingly, it is worth noting that PDW was a significant indicator of severe cases of COVID-19. PDW is utilized to depict the distribution of PLT volume, and when PLT is excessively consumed, the bone marrow produces abundant immature PLT that is larger than mature PLT. PDW is also significantly associated with sepsis and other severe illnesses, which is closely linked to poor COVID-19 outcomes and death (31, 32). In this study, the severe group showed a larger PDW, with a mean of 17.35 versus 16.88, which was significantly different from the non-severe group (p =0.001).

During the stage of systemic inflammation in COVID-19, there is a significant increase in inflammatory biomarkers like IL-2, IL-6, and CRP, which are dramatically enhanced. This stage represents the most severe manifestation of cytokine storms, and excessive inflammation may lead to multiple organ dysfunction (33–35). According to recent research, IL-6 has been identified as a predictive factor for the early detection of COVID-19 patients who are at a heightened risk of experiencing worsening disease progression (36, 37). Elevated IL-2 levels observed in individuals with COVID-19 could potentially suggest the activation of T cells (38). In this study, the levels of IL-2, IL-6, and CRP in the severe group were significantly higher than those in the non-severe group (p<0.01). Research has indicated that individuals with severe COVID-19 often experience a high prevalence of coagulation abnormalities (39). Recent pathological results show that immune thrombosis in these patients gathers inflammatory cells such as lymphocytes and neutrophils, and the immune thrombosis can develop into serious complications, which are strongly associated with the severity of the disease and mortality rates (40–42). In the present study, the levels of DD and PT were markedly elevated in severe patients than in non-severe individuals (p<0.01), consistent with the findings of Huang et al. (41) and Wang et al. (43). Elderly patients exhibit a continual inflammatory response and compromised coagulation after being infected with SARS-CoV-2, as evidenced by elevated levels of coagulation and inflammatory markers. Severe patients exhibited a greater degree of inflammation.

Of all antibodies against post-infection immunization, the IgG antibodies were the most prominent signature. This antibody not only marks the later stages of infection but also remains in the body for at least 6 months (44). IgG 1 is the most common IgG subtype, and viral infection usually induces both IgG 1 and IgG 3 (45). There were a few studies that reported the emergence of IgM and IgG antibodies when the SARS-CoV-2 virus invaded and suggested the application of serologic tests in the diagnosis of COVID-19 (46, 47). However, there is limited documentation regarding the IgG subtypes that are generated following SARS-CoV-2 infection (48, 49). According to Husain et al. (39), it was discovered that there could be a prevalence of abnormalities in IgG subtypes among severely ill COVID-19 patients, which should be further examined, as it could serve as an indicator of disease severity and a potential target for therapy. In the present study, significant variations in IgG subcategories were observed between healthy individuals and elderly COVID-19 patients (p<0.05). The study included IgG Sum/IgG 1 in the LASSO regression, which indicated that its predictor of COVID-19 severity in elderly patients outperformed individual IgG subtypes. Another important finding of the study was that IgG Sum/IgG 1 showed extremely significant differences between the two groups compared to IgG subtypes alone (p =0.009). The data show that the IgG 1 level of severe patients is significantly lower than that of non-severe patients (median, 5,614.50: 6,645.00), and IgG3 levels are higher than non-severe patients (median, 247.50: 203.00), and this variation could be attributed to the source of the samples and the clinical treatment received, indicating distinct *in vivo* IgG subtypes among different populations, even when they are infected by the identical pathogen.

Nomograms were used to assess their capability to predict the likelihood of severe illness upon admission in a number of studies (50, 51). No study, however, has investigated the potential of novel factors related to IgG subtypes in elderly patients with COVID-19. Although Sun et al. (14) established a model for predicting severe COVID-19 (sensitivity=100%, specificity=88.89%), it only included IgA, NE #, and EO # while neglecting IgG subtypes. In the present study, a predictive model of elderly patients was constructed with AUC=0.824, which is higher than the AUC=0.800 in the study by Zeng et al. (13). However, this study included specific laboratory indicators like IL-2 and IgG subtypes, which included new indicators of the immune response. Based on our current understanding, this model is the initial attempt to forecast the severity of elderly COVID-19 patients based on IgG subtypes.

## Limitations

There were some limitations in this study. First of all, this study consisted of only 103 elderly individuals diagnosed with COVID-19. The sample size of 103 patients may be considered small. In further research, we will expand more participants and diversify the sample size from multiple sources to improve the generalization and performance of the model in different settings. Second, this model was built and verified using data from China. Patients from diverse nations and races in future studies need to be included to confirm the results. Moreover, there may be some inevitable bias, and clinicians’ assessment of disease severity may be subjective, potentially leading to some overlap between the severity groups. Finally, the present study might have resulted in variations in the outcomes of elderly COVID-19 patients from different hospitals at distinct time points during the peak of the COVID-19 outbreak in the current year. In the future, we will optimize the model and correct the defects of our model based on the present study.

## Conclusion

In conclusion, a model based on machine learning for predicting the severity of COVID-19 was constructed. Four indicators (age, DD, IL-2, and IgG Sum/IgG 1) are filtered to construct the model. Five machine learning models (XGBoost, AdaBoost, SVM, logistic regression, and random forest) were used on the same dataset to predict the severity of elderly COVID-19 patients. The logistic regression model demonstrated the best prediction performance among them. In addition, the present study conducted external validation of the model using data from two other centers. This model demonstrates excellent discrimination and calibration, making it readily applicable in clinical practice, may predict outcomes as early as admission, and could assist clinicians in estimating COVID-19 severity and improving elderly patient outcomes. In further research, we will collect further data and conduct a multi-center study to enhance the generalization of the model. In addition, we are working on developing an online website or an applet plugin based on our model to facilitate its use by clinical practitioners. This will provide an efficient and user-friendly interface for doctors to input patient symptoms and get predictive results from the model.

## Data availability statement

The original contributions presented in the study are included in the article/supplementary material. Further inquiries can be directed to the corresponding author.

## Ethics statement

The studies involving humans were approved by Ethics Committee of The First Affiliated Hospital of Zhejiang Chinese Medical University. The studies were conducted in accordance with the local legislation and institutional requirements. The participants provided their written informed consent to participate in this study.

## Author contributions

ZZ: Investigation, Writing – review & editing. YQ: Formal Analysis, Writing – original draft. YMY: Data curation, Methodology, Writing – review & editing. YY: Project administration, Writing – review & editing.
